# Fungal Parasitism: Life Cycle, Dynamics and Impact on Cyanobacterial Blooms

**DOI:** 10.1371/journal.pone.0060894

**Published:** 2013-04-12

**Authors:** Mélanie Gerphagnon, Delphine Latour, Jonathan Colombet, Télesphore Sime-Ngando

**Affiliations:** LMGE, Laboratoire Microorganismes: Génome et Environnement, UMR CNRS 6023, Clermont Université, Université Blaise Pascal, BP 80026, Aubière, France; University of New South Wales, Australia

## Abstract

Many species of phytoplankton are susceptible to parasitism by fungi from the phylum Chytridiomycota (i.e. chytrids). However, few studies have reported the effects of fungal parasites on filamentous cyanobacterial blooms. To investigate the missing components of bloom ecosystems, we examined an entire field bloom of the cyanobacterium *Anabaena macrospora* for evidence of chytrid infection in a productive freshwater lake, using a high resolution sampling strategy. *A. macrospora* was infected by two species of the genus *Rhizosiphon* which have similar life cycles but differed in their infective regimes depending on the cellular niches offered by their host. *R. crassum* infected both vegetative cells and akinetes while *R. akinetum* infected only akinetes. A tentative reconstruction of the developmental stages suggested that the life cycle of *R. crassum* was completed in about 3 days. The infection affected 6% of total cells (and 4% of akinètes), spread over a maximum of 17% of the filaments of cyanobacteria, in which 60% of the cells could be parasitized. Furthermore, chytrids may reduce the length of filaments of *Anabaena macrospora* significantly by “mechanistic fragmentation” following infection. All these results suggest that chytrid parasitism is one of the driving factors involved in the decline of a cyanobacteria blooms, by direct mortality of parasitized cells and indirectly by the mechanistic fragmentation, which could weaken the resistance of *A. macrospora* to grazing.

## Introduction

Most parasitic zoosporic true fungi found in lakes belong to the phylum Chytridyomycota (i.e. chytrids) [1]. Although significant progress has recently been made with new approaches designed to assess their diversity and potential functions [2], [3], [4], [5], [6], [7], these fungi are still poorly studied in pelagic systems. Furthermore, little is known about their dynamics in freshwater food webs. Large and/or colonial phytoplankton species are particularly susceptible to chytrid parasitism [8], [9], and are known to transfer matter and energy to zooplankton *via* grazing of fungal zoospores, through the so-called ‘mycoloop’ [9]. The most frequently studied models of fungal parasites of phytoplankton are those which infect eukaryotic hosts, primarily diatoms and chlorophytes, and declines in the size of blooms can be accelerated by parasitism [10], [11], [12], [13], [14], [15].

Most of the studies on the causes of filamentous cyanobacterial bloom decline have primarily considered physico-chemical factors, such as temperature, light, and the availability of nutrients [16], [17], [18], [19], [20]. Predation has been studied, but it has a weak impact on population densities of colonial or filamentous cyanobacteria [21], [22], [23]. Several studies have also investigated the impact of viruses on marine and freshwater cyanobacterial populations, clearly highlighting the effects of viruses on cyanobacterial bloom collapse [24], [25], [26], [27], [28], [29].

Despite its potential roles in the decline of filamentous cyanobacteria, chytrid parasitism remains poorly studied. The few published studies on cyanobacterial-chytrid interactions were based on laboratory experiments with a focus on taxonomic description of fungi and host-parasite interactions, or on field studies, the durations of which largely exceed the generation times of both hosts and parasites [6], [30], [31], [32]. To overcome the bias of cultivation conditions, and their putative alteration of host-parasite interactions [33], and to provide an accurate host-parasite couple dynamic, we surveyed a recurrent chytrid-cyanobacterium assemblage in a productive lake using a fine resolution sampling strategy over the entire bloom period of the filamentous heterocystous cyanobacterium *Anabaena macrospora.* Our specific objectives were to (i) analyze the dynamics of parasites and describe the different stages of their life cycles using direct microscopic observations, (ii) determine the infective strategies of chytrids in the different cellular niches (i.e. akinetes, vegetative cells) offered by the host, and (iii) infer the putative role of fungal parasitism in the decline of cyanobacterial blooms.

## Materials and Methods

### Study Site and Sample Collection

Samples were collected in Lake Aydat (45°39′48′′N, 002°59′04′′E), a small eutrophic lake (Zmax = 15 m, surface area = 60 ha) with a large catchment area (3×10^4^ ha) located in the French Massif Central, where recurrent blooms of cyanobacteria occur in late summer and early autumn. Samples were collected every 3 days from the 6^th^ of September to the 30^th^ of October 2010, corresponding to the seasonal bloom of *Anabaena macrospora*. Samples were taken from the center of the lake at the point of maximum depth. To comprehend the vertical distribution of cyanobacteria and associated chytrid parasites, samples were collected at two different depths during each sampling date. The first was at a constant discrete subsurface depth (0.5 m) and the second varied from 1 to 4 m and depended on the maximum depth of fluorescence (MF) determined *in situ* from the vertical pigment profiles obtained by a BBE Fluoroprobe® (Moldaenke, Germany). 20 liters were sampled using an 8-L Van Dorn bottle. To eliminate the metazoan zooplankton, collected samples were immediately prefiltered through a 150 µm-pore-size nylon filter, poured into clean transparent recipients, and then transferred immediately to the laboratory for processing. The fraction >150 µm was verified to ensure that no cyanobacterial cells were retained.

No specific permits were required for the described field studies, as the location is not privately-owned or protected in any way, and the field studies did not involve endangered or protected species.

### Physico-chemical Parameters

Water transparency was measured *in situ* with a Secchi-disk. Temperature and dissolved oxygen profiles were obtained using a multiparameter probe ProOdO™ (Ysi, Germany). For determination of nitrate, ammonium and orthophosphate, 3 replicates×50 ml of sampled waters were filtrated through 0.2 µm syringe filter and stored frozen at −20°C until analysis using spectroquant reagent standard kits (Merck, Germany).

### Phytoplankton Community

For phytoplankton analyzes, triplicate 180 ml of raw samples were fixed with Lugol’s iodine and 5 to 20 ml subsamples (for each replicate and depending on the phytoplankton density) were settled overnight in counting chamber and cells counted under an epifluorescence microscope (Zeiss Axiovert 200 M) according to the classic Utermöhl method. At least 400 cells were counted on at least 30 randomly selected optical fields. Phytoplanktonic cells were identified, at times up to the species level, using morphological taxonomic keys known from references books (e.g., [34], [35]). The targeted cyanobacterium *A. macrospora* [misidentified as *Anabaena flos aquae* in Rasconi *et al.* (2012)], was quantitatively analyzed. The two species were distinguished on the basis of trichomes and the form and size of akinetes. *A. macroscopora* exhibits a straight single trichome and rounded akinetes (size 16–18 µm wide, 17–26 µm long) while *A. flos aquae* has a coiled trichome that forms a solitary or more usually an entangled twisted mass, with cylindrical or slightly ellipsoid and often slightly bent akinetes (size 6–13 µm wide, 20–50 µm long) [36]. The total numbers of *Anabaena macrospora* cells (vegetative cells and akinetes) and filaments in the samples were recorded and inspected for chytrid infection.

### Chytrid Parasitism

For chytrid infection parameters, samples were treated using the size-fractionated community method developed by Rasconi *et al*. [5]. Briefly, 18 L of sampled water was concentrated on 25 µm pore size nylon filter. Large phytoplankton cells (≥25 µm), including the filamentous cyanobacteria *A. macrospora*, were collected by washing the filter with 0.2 µm-pore-size-filtered lake water, fixed with formaldehyde (2% final concentration), and an aliquot of 195 µl was stained for the chitin cell wall characteristic of fungi. We used the fluorochrome calcofluor white (CFW, C_40_H_44_N_12_O_10_S_2_, excitation wavelength 440 nm; emission wavelength 500–520 nm, Sigma catalog no F3543) at a concentration of 2.5% (vol/vol), from a stock solution [5]. Staining lasted at least 10 minutes before mounting between glass slides and cover slips and observation under the epifluorescence microscope (Zeiss Axiovert 200 M) using UV light excitation at ×400 magnification. We systematically inspected 50 filaments comprising 780 to 2085 individual cells of *A. macrospora* to determine (i) the number of infected and non-infected cells and filaments, and (ii) the number of infected and non-infected cells for each filament. In addition, a minimum of 100 akinetes were inspected for the number of infected and non-infected akinetes. The original triplicates of each sample collected were analyzed. Infection parameters were calculated according to the formula proposed by Bush *et al.* [37]. These parameters include the prevalence of infection (Pr), i.e., the proportion of individuals in a given population with one or more fixed sporangia or rhizoids, expressed as Pr (%) = [(*N*i/*N*)×100], where *N*i is the number of infected host cells (or filaments), and *N* is the total number of host cells (or filaments). We distinguished four types of infection prevalences: (i) PrC or the percentage of infected *A. macrospora* cells calculated from the total number of cells in the whole population; (ii) PrF or the percentage of infected filaments calculated from the total number of filaments, (iii) PrCF or the percentage of infected cells within those filaments which were infected, and (iv) PrAK or the prevalence of infection for akinetes. Moreover, the entire life cycle of chytrid species was described from microscopic observations and 6 tentative life stages were delineated based on phenotypic characteristics given by Canter [38], from the younger to the more mature stage. The stage in the life cycle for each sporangium observed in our samples was assigned according to our model.

### Statistical Analysis

One way analysis of variance (ANOVA) was applied to test the differences between the sampling depths for the dynamics of *A. macrospora* cells and the related prevalence of infection. When significant differences were noted, a post hoc comparison (Tukey’s Honestly Significant Difference [HSD], α = 0.05) was used. Spearman’s linear correlation was used to test empirical relationships between the variables under study. All statistical analyses were performed using Past software available at http://folk.uio.no/ohammer/past/ [39].

## Results

### Physico-chemical Environment

The sampling period corresponded to the seasonal cooling phase of the lake, with a weak stratification for the first sampling date (19 and 17.6°C at 0.5 m and maximum fluorescence (MF) depth, respectively) that disappeared from the second date towards the end of the sampling period (10°C in both depths), although an anomaly was observed in early October (Fig. S1). The MF depth naturally corresponded to the depth of maximum dissolved oxygen. The concentration of nutrients (nitrogen and phosphorous) showed great temporal variations (Figure S1), but no correlation was noted between nutrient concentrations and cyanobacteria abundance.

### Phytoplankton Abundance

**Figure 1:**
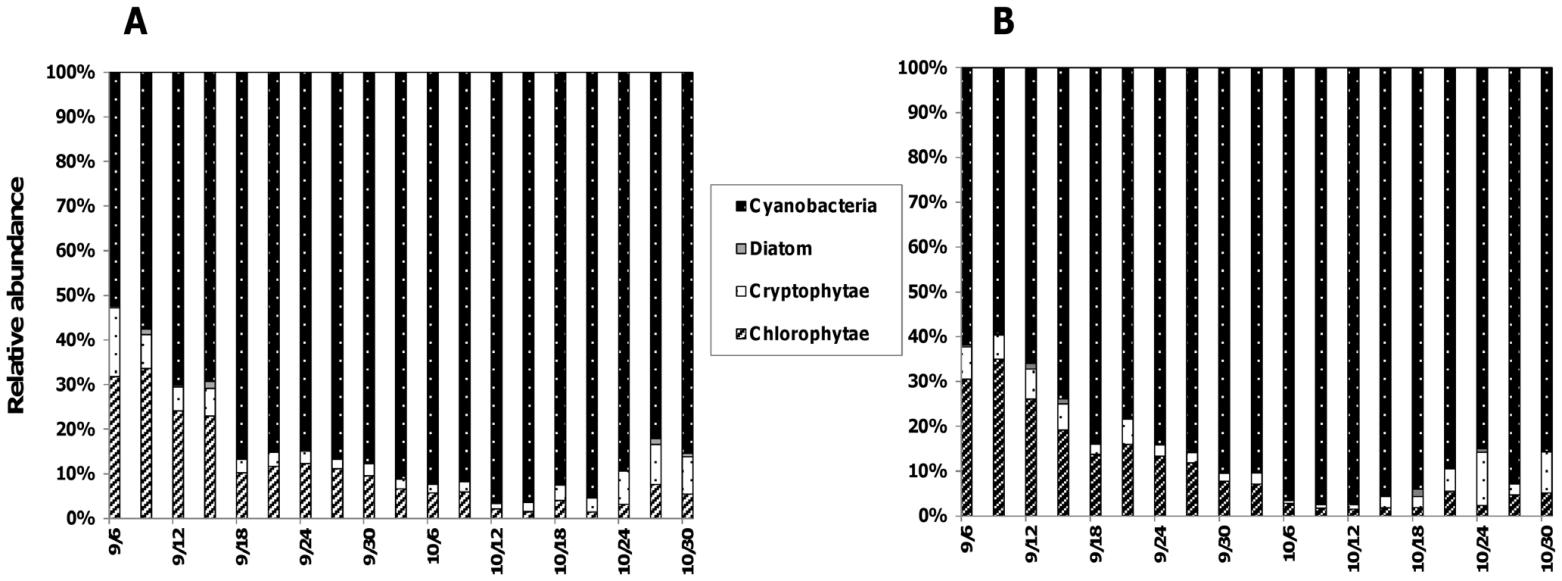
Composition of phytoplanktonic community. Relative contributions of the different taxonomic groups within the phytoplanktonic community at 0.5 m (A) and in the depth of maximum chlorophyll, MF (B) in Lake Aydat, September 6^th^ to October 30^th^ 2010.

Our sampling period corresponded to the seasonal bloom of the targeted cyanobacteria *A. macrospora*, which largely dominated the phytoplankton community in the lake. Their abundances at the two sampling depths (mean for triplicates ± SD) fluctuated from 2.74±0.1 to 24.7±0.7×10^6^ cells.l^−1^ and from 4.4±0.1 to 38.5±0.6×10^6^ cells.l^−1^ at 0.5 m and MF depth, respectively. At the beginning of the sampling period (i.e. first half of September), *A. macrospora* accounted for 52 and 62% of the total phytoplankton abundance at 0.5 m and MF, respectively, values which increased to reach 96% and 98% at the bloom maximum in mid-October (Fig. 1). Accompanying phytoplankton species mainly belonged to chlorophytes (1–34% for the two sampled depths), cryptophytes (1–15 and 0.8–7% at 0.5 m and MF, respectively), and diatoms (0.1–1% for the two sampled depths).

**Figure 2:**
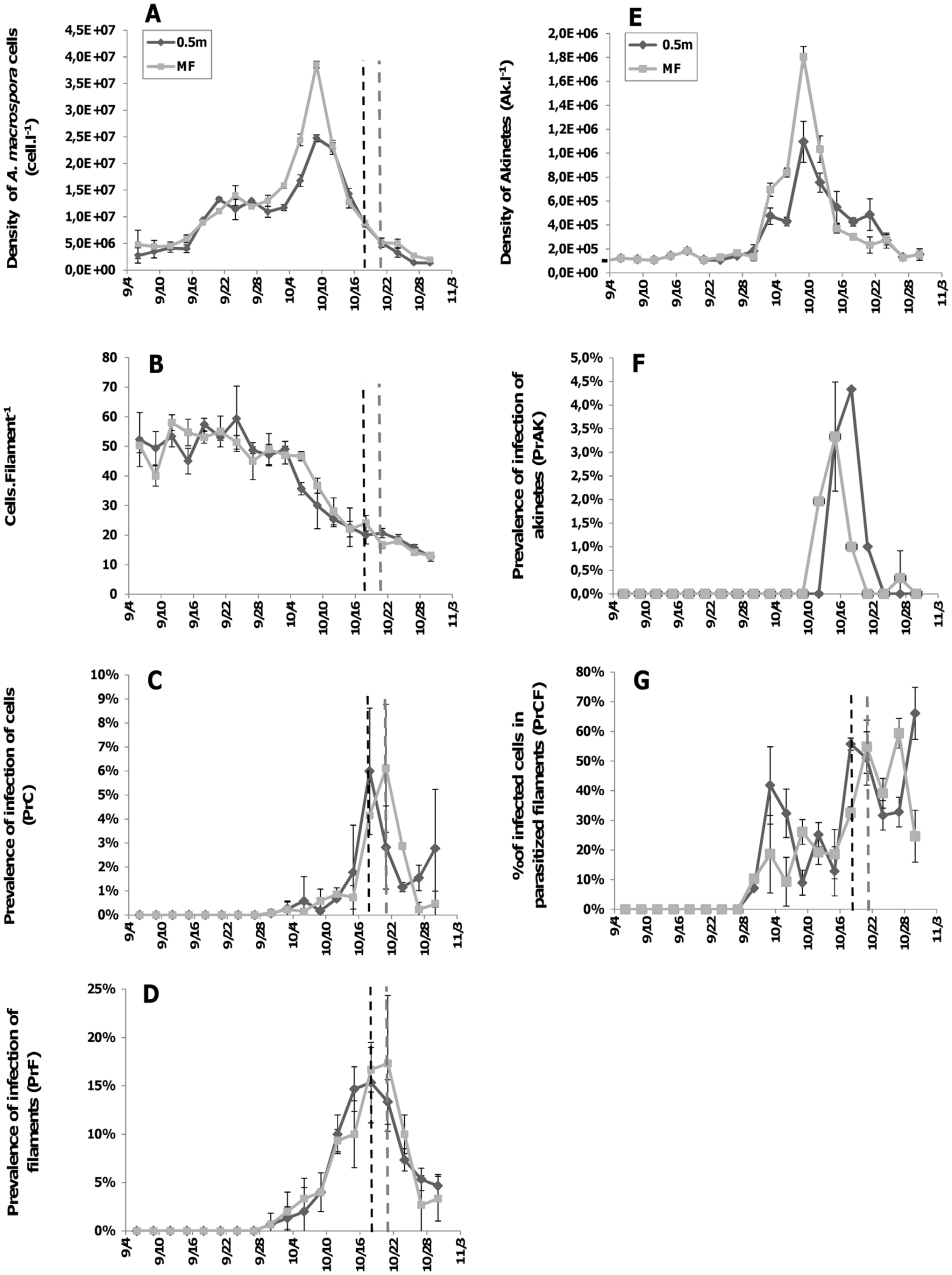
Dynamics of host and chytrid parasitism parameters. Changes in the density of *Anabaena macrospora* total cells (A), cells per filament (B) and akinetes (E), and in the prevalences of infection of *A. macrospora* cells (C, PrC), filaments (D, PrF), akinetes (F, PrAK), and of cells in infected filaments (G, PrCF) at 0.5 m and in the depth of maximum chlorophyll (MF) in Lake Aydat, September 6^th^ to October 30^th^ 2010. Vertical lines mark the transition point between the increasing and the decreasing phases in the prevalence of infection of filaments (PrF), at 0.5 (dark dashed line) and MF (grey dashed line) depths.

The temporal changes in the abundances of *A. macrospora* were quite similar for the two depths sampled and exhibited 4 different growth phases: a relatively stagnant phase during the first half of September (mean growth rate, µ = 0.03 d^−1^ for the two sampling depths), a moderate increasing phase during the second half of September (µ = 0.10 d^−1^), a more rapidly increasing phase during the first half of October when an apparent difference was noted between the two depths (µ = 0.13 and 0.15 d^−1^ at 0.5 m and MF, respectively), and a decreasing phase towards the end of the study (µ = −0.14 d^−1^ for the two sampling depths). As a consequence, the numerical abundances were similar for the two sampled depths, except during the rapidly increasing phase (i.e. from October 3^rd^ to 9^th^) when they were significantly (p<0.02) higher in the MF depth (maximum density 3.9±0.06×10^7^ cells.l^−1^) compared to 0.5 m (2.5±0.06×10^7^ cells.l^−1^) (Fig. 2A). We observed that the size of the filaments of *A. macrospora* shifted significantly (p<0.05) below 40–60 cells.filament^−1^ from the 3^rd^ of October (i.e. the starting point of the rapidly increasing bloom phase) to the end of the bloom (Fig. 2B).

As with the vegetative cells of *A. macrospora*, the abundance of akinetes rapidly increased and differed from one depth to another (p<0.05) during the first half of October, and then decreased towards the end of the sampling period (Fig. 2E). Maximum abundances recorded on October 9^th^ reached 1.09±0.01 and 1.81±0.08×10^6^ akinetes.l^−1^ at 0.5 m and MF depths, respectively (Fig. 2E).

### Reconstructing the Life Cycle of Chytrids

**Figure 3:**
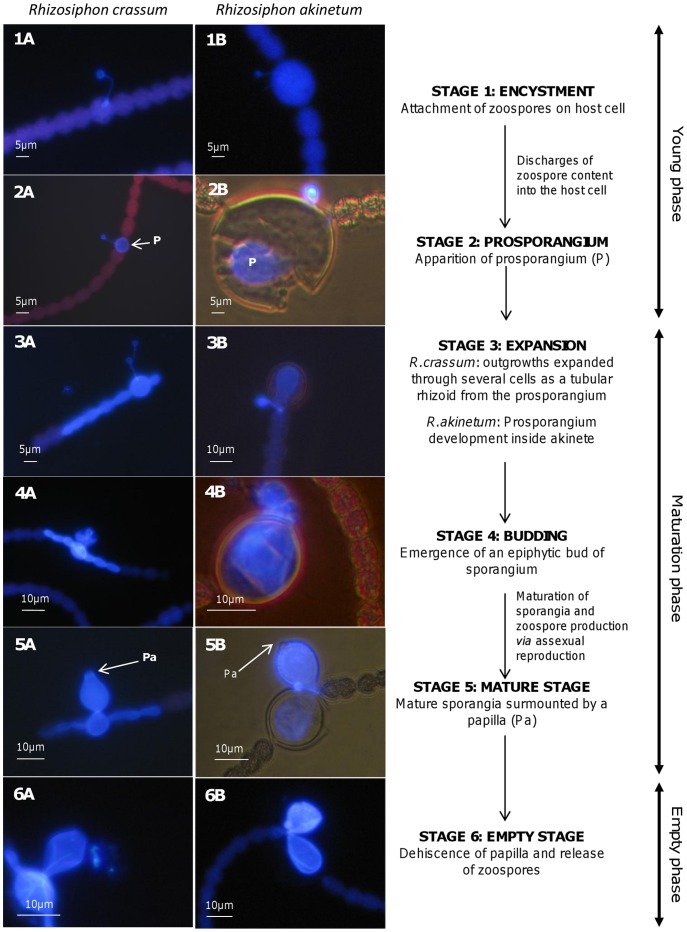
Life cycles of the two chytrid species. The six different life stages of the two chytrid species, *Rhizosiphon crassum* (A) and *Rhizosiphon akinetum* (B) parasitizing the cyanobacterium *Anabaena macrospora* from the productive Lake Aydat: Stage1 : Encystment; Stage 2 : Prosporangium; Stage 3 : Expansion stage; Stage 4 : Budding; Stage 5 : Mature stage; Stage 6 : Empty stage. The six life stages were grouped into three different phases: Young phase (Stages 1 and 2), Maturation phase (Stages 3, 4 and 5) and Empty phase (Stage 6). Prosporangium (P) and Papilla (Pa).

Based on the morphology of the sporangium and on the type of host cells, we were able to identify two species of chytrid parasites of *A. macrospora* both belonging to the same genus, *Rhizosiphon*. *R. crassum* infected both vegetative cells and akinetes by developing a tubular rhizoid system (Fig. 3_1–6A_), while *R. akinetum* infected only akinetes (Fig. 3._1–6B_). We distinguished 6 tentative stages in the life cycle for these two chytrids, based on the description given by Canter [38]: Encystment, Prosporangium, Expansion, Budding, Mature, and Empty stages.

Stage one, Encystment, is represented by a zoospore that has just penetrated the mucilage of a living host cell with a fine thread (Fig. 3._1A_, 3._1B_). The second stage is the prosporangium. In the prosporangium stage the contents of the zoospore are discharged into the host cell, resulting in a globose structure known as the prosporangium (Fig. 3._2A_, 3._2B_). In the two chytrid species these first two stages can only infect a single host cell and thus were grouped together as “young phase” in the dynamics of life stages (see below). From the prosporangium, outgrowths, characteristic of tubular rhizoids, expands through several cells. This is the third stage: Expansion (Fig. 3._3A_). The life cycle continues on to the Budding stage (the fourth stage) with the emergence of an epiphytic bud (Fig. 3._4A_). This bud develops into a flask-shaped sporangium in which zoospore production occurs (asexual reproduction). It is surmounted by a gelatinous papilla typical of the mature sporangium of *R crassum* and forms the fifth stage: the Mature stage (Fig. 3._5A_). These three later stages (Expansion, Budding, and Mature stage) were grouped together in the dynamics of life stages into the “Maturation phase”. The sixth and final stages of the life cycle for *R crassum* is the Empty stage (and phase) that forms after deliquescence of papilla and release of zoospores (Fig. 3._6A_). In *R akinetum*, Encystment (Fig. 3._1B_), Prosporangium (Fig. 3._2B_), Expansion (Fig. 3._3B_), Budding (Fig. 3._4B_), Mature (Fig. 3._5B_) and Empty stages (Fig. 3._6B_) were also observed, except that there is no tubular rhizoidal system because the infection is restricted to akinetes (Fig. 3._3A_ vs. 3._3B_).

### Temporal Changes in the Life Stages of *R. crissum*

**Figure 4:**
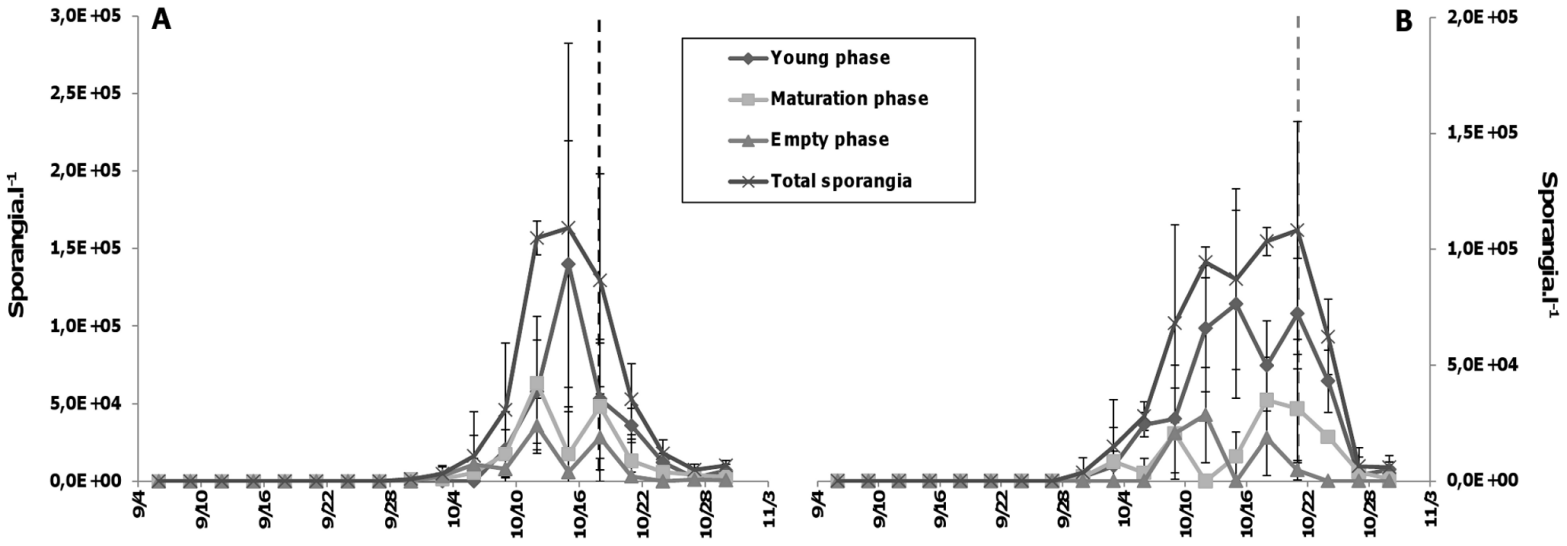
Dynamics of the different phases of life cycle of *Rhizosiphon crassum*. Dynamics of the three different phases of the life cycle of the chytrid *Rhizosiphon crassum* infecting the cyanobacterium *Anabaena macrospora* at 0.5 m (A) and in the depth of maximum chlorophyll (MF) (B) in Lake Aydat, September 6^th^ to October 30^th^ 2010. The three phases regroup the six stages of life as above: Young phase (Stages 1 and 2), Maturation phase (Stages 3, 4 and 5) and Empty phase (Stage 6) (see the main text for details). Vertical lines mark the transition point between the increasing and the decreasing phases in the prevalence of infection of filaments (PrF), at 0.5 (dark dashed line) and MF (grey dashed line) depths.

Because the abundance of *R. akinetum* was very low (data not shown) and the first life stages of the two chytrid species were quite similar and difficult to differentiate based on their morphology, the dynamics of chytrid life stages is reported only for *R. crassum* infecting vegetative cells (Fig. 4). No differences have been reported for all stages at the two sampled depths (Fig. 4A vs 4B). Independently of the depth, abundance of sporangia increased significantly from the 3^rd^ of October to reach its maximum values, approximately 10 fold increase, on the 15^th^ and the 21^st^ of October (1.6±1.1×10^5^ and 1.08±0.4×10^5^ sporangia.l^−1^, at 0.5 m and MF, respectively.).

At 0.5 m, the increase in total sporangia was related to (i) the increase in young life stages which peaked on October 15^th^ and represented 85% of total sporangia, (ii) followed by the increase in the maturation life stages plus empty sporangia between October 15^th^ and 18^th^ (Fig. 4A). In the same way, the increasing phase of sporangia was mostly due to the young life stages which averaged 67% of total sporangia on October 21^st^ at MF depth, enhanced by an increase in the maturation life stages plus empty sporangia between October 15^th^ and 18^th^ (Fig. 4B). Interestingly, for the two sampled depths, we noted that 3 days before each abundance peak of young chytrid life stages, empty sporangia and sporangia involved in the maturation process showed an increase, with their maximum abundances being systematically lower than those of young sporangia (Fig. 4).

### Prevalence of Infection

The infection of *A. macrospora* vegetative cells (PrC) by *R. crassum* lasted about 30 days, starting the 30^th^ of September and involved <1% of *A. macrospora* cells in the two depths. PrC then increased significantly (p<0.001) to reach a maximum of about 6% on the 18^th^ of October at 0.5 m and on the 21^st^ of October at the MF depth, i.e. during the declining phase of the cyanobacterial bloom. From these dates, PrC decreased significantly (p<0.005) until the end of our survey (Fig. 2C). The infection period of akinetes by *R. akinetum* lasted about 2 weeks, roughly between October 12^th^ and 24^th^, with maximum prevalence of 4.3 and 3.5% at 0.5 m and MF depths, respectively (Fig. 2F).

Changes in the infection prevalence in the filaments (PrF, i.e. percentage of infected filaments calculated from the total number of filaments) were similar to those in the whole *A. macrospora* population (i.e. PrC), including the maximum values which were observed on October 18^th^ at 0.5 m and on October 21^st^ at MF (Fig. 2d). Nevertheless, the values of the infection prevalence for filaments were higher than those obtained for the whole population, and reached 15±3.1% at 0.5 m and 17±4.2% at MF. PrF then decreased rapidly and significantly (p<0.05) towards the end of the sampling period (Fig. 2D).

Within a filament, the percentage (0 to 66±9%) of infected cells to the total cell number, i.e. PrCF, were in the same range and fluctuated similarly in both depths (Fig. 2G). Fluctuations in PrCF were consistent for the two depths but, overall, roughly increased during the whole infection period, i.e. from September 30^th^ to October 30^th^. However, when averaged PrCF values in Figure 2G for two critical periods, i.e. the increasing and the decreasing periods for both PrC and PrF (i.e. Fig. 2C,D), the mean PrCF values for the increasing period (21±11 and 19±6% at 0.5 m and MF, respectively) were significantly (p<0.05) lower compared to those (47±10 and 45±12%) calculated for the decreasing period. Clearly, the number of infected cells within those filaments, which were infected, was significantly higher during the declining phase of *A. macrospora* bloom, compared to the growth phase.

## Discussion

### General Considerations

This study constitutes an original report on fungal parasitism associated to a complete cyanobacterial bloom event in a natural aquatic ecosystem. Our data contribute significantly to a better understanding of both the life cycle of chytrids in natural environment and their infection strategies. Although our species identification was solely based on morphological traits [38], [40], the data in our study highlight that, at the natural community level, a single cyanobacterial host species (*Anabaena macrospora*) can offer different cellular niches for two parasites in the Chytridiomycota (i.e. chytrids): *Rhizosiphon crassum* and *Rhizosiphon akinetum*. Similarly, a previous study has shown that another freshwater *Anabaena*, *A. smithii*, could also be parasitized by two different chytrid species which were able to infect akinetes and heterocysts. However, the identity of the chytrids in the study by Takano *et al.* [32] in Lake Shumarinai, Hokkaido, Japan, was not determined. In general, *Rhizosiphon* species are well known as chytrid parasites of cyanobacteria and, more particularly, of the genus *Anabaena* [30], [40], [41]. The possibility that the two species of *Rhizosiphon* infecting the same host represent cryptic forms of the same species, e.g. based on the as yet unavailable molecular sequences, remains an interesting open question.

### The Duration of the Life Cycle of Chytrid Parasites from a Field Study Point of View

We have identified six different phenotypic life stages [38] for *R. crassum* and *R. akinetum* due to a high sampling resolution coupled to an improved staining technique [5] and have provided a tentative full description for *R. crassum*. We have grouped these six stages in the life cycle into three phases (young, maturation and empty phase) corresponding to the growth phases (i.e. encystement, germination, growth and maturation) known from the general life cycle of the Chytridiomycota. In our field survey, the young phases were followed within a period of three days by empty phases, and so we were able to infer that the complete life cycle of *R. crassum* lasted about 3 days in natural conditions. Cultivation experiments could give more precise information on optimal conditions of growth. Nevertheless, our field results seem to be in agreement with results obtained for other parasitic chytrid species maintained in laboratory conditions. Indeed, Bruning and Ringelberg [42] calculated that *Rhizophydium planktonicum*, a typical chytrid parasite of diatoms, accomplished a full life cycle for about 2 days in optimal conditions. Recently, Berger *et al.* [43] described the life cycle of *Batrachochytrium dendrobatidis*, one of the most deadly contemporary skin disease agent that drives the decline of amphibian populations worldwide, and suggested that the time for completion of the life cycle was between 3 and 5 days. From these comparisons, it is thus likely that our sampling resolution was high enough to determine the generation time of the parasitic chytrids under study during the seasonal bloom of *A. macrospora*.

### One Genus, Two Species, and Different Strategies for Infection

The main differences in strategies used by the two chytrid species were the type of targets (host cells) in *A. macrospora* filaments, and the methods of parasitic exploitation of these cells. Infectivity of *R. crassum* was observed throughout the whole filament, with rhizoids crossing through both vegetative cells and akinetes, whereas that of *R. akinetum* was highly specific and restricted to akinetes. These differences suggest the co-occurrence of different infectivity strategies, depending on the type of cellular niches offer by the hosts and, we suspect, on the availability of energy required for the parasite development. Because of its capacity to infect several cells at the same time, *R. crassum* could be considered as having access to more energy than *R. akinetum*. However, there might be an energetic advantage to infect akinetes. Compared to vegetative cells, akinetes are known to contain approximately 2-fold more carbon [44], and 16-fold more glycogen which constitutes the prime energy reserves of zoospores for dispersal [45], [46]. This indicates that the zoospores of *R. akinetum* could have access to the same or even more energy than those of *R. crassum*, in spite of the restricted to akinetes. However, akinetes were not present during the entire bloom event and presented a systematically lower abundance than vegetative cells (i.e. 10 to 150 fold). Because chytrid infection is generally a host density-dependent process [8], we consider that the ability of *R. crassum* to infect both vegetative cells and akinetes could be an advantage in our case study. The type of cells within the same host species could thus influence the process of chytrid infection in natural communities of filamentous cyanobacteria, depending on the resource availability for spreading infection and prevalence.

The time necessary to complete the life cycle of zoosporic true fungi is short and their dissemination phase is known to be highly dependent on the host density [12], [47], [48], [49]. In our case study, infections seemed to start from a minimal host density threshold of 1.5×10^7^ vegetative cells liter^−1^ and 1.8×10^6^ akinetes liter^−1^ for *R. crassum* and *R. akinetum*, respectively (Fig. 2). This indicates that chytrid infection could be promoted at relatively low host densities in natural conditions [50], [51]. Nevertheless, detailed analysis of chytrid infection revealed that the maximum prevalence was mainly due to the increase of infected cells within the already infected filaments (Fig. 2D). These results imply that, at the end of the bloom event, some trichomes could be intensely infected while others were completely healthy, which was observed during the end of our survey, suggesting a coexistence of resistant and susceptible *A.macrospora* filaments to the fungus attacks. It is well known that inside a host population, some genotypes are more susceptible to parasitism than others [52], [53], [54]. Recently, Sonstebo & Rohrlack [6] demonstrated a close relationship between genotypes, chemotypes and the severity of chytrid infection for strains of the cyanobacteria *Planktothrix*. Furthermore, other authors have indicated chemotactism as one mechanism to explain the attraction between hosts and chytrids in environmental samples [55], [56], [57]. In the case of decreasing host cell density such as during the late bloom phase when the maximum prevalence was recorded in our study, we suggest that newly produced zoospores could be more attracted by the chemical cues from the nearby non-infected cells located within infected filaments, compared to those cells in non-infected filaments located too far away for an efficient chemotactic detection. This may help explain the paradox of the co-occurrence of low cellular density of *A. macrospora* and low infection prevalence of filaments, with a high number of infected cells within parasitized filaments at the end of the bloom (Fig. 2A,E,F). This could also be explained by a simple opportunistic development (i.e. of both parasites and saprotrophs) on moribond host individuals in the declining bloom phase. However chytrid infection started when cyanobacteria population was in a rapidly growing phase characterized by (i) an enhanced increase in cellular density, (ii) high chlorophyll content as attested by high autofluorescence of cells (Fig. 3._2A_), and (iii) high proportion of cells located above the limit of euphotic layer for the two sampling dephts. These conclusions tend to confirm previous observations that chytrids parasitize healthy hosts and thus impact directly the dynamics of cyanobacterial populations [8], [58], [59], [60].

### Parasitism by Chytrids has Direct and Indirect Effects on Filamentous Cyanobacterial Blooms

Parasitic chytrids derive their growth energy from the host cells, a situation that may trigger the death of the latters. Parasites can also reduce the fitness of their host, or allow infected hosts to remain strong competitors [61]. These direct effects of parasites could vary greatly with space and time. In this study, the infection prevalence peaked at 20% of total filaments, which is considerably lower than the value of 98% reported in 2007 in the same lake for the same host species [62]. In Shearwater Lake, Wiltshire, United Kingdom, Sen [31] also reported a significant difference in the severity of chytrid infection between successive years, during the summer-autumn development of the cyanobacteria *Microcystis aeruginosa.* Nevertheless, in these last two studies which centered on the host genera *Anabaena* and *Microcystis*, abiotic factors and host density were quite similar and failed to empirically explained the interannual differences in the chytrid infectivity during seasonal cyanobacterial blooms. It was reported that cyanobacterial blooms often are the result of the growth of a few dominant genotypes [63], which may change from year to year in a same lake [64]. Sonstebo and Rohrlack, (2011) have emphasized that chytrid virulence was strain dependent. Thus, interannual shift in genotypic composition of host population could lead to different sensitivities to parasitism. The sensitivity to fungal parasitism for the dominant genotype can be quite high and contribute significantly to accelerate the decline of the blooms with liberation of niches for other species [65] or, conversely, can be low and weaken the top-down effect of chytrids on a restricted fraction of cyanobacterial population.

Besides their role in maintaining phytoplankton diversity, chytrids also infect resistance forms (i.e akinetes) during the pelagic phase of cyanobacteria. Akinetes are key elements that promote the seasonal germination of cyanobacterial filaments when favorable conditions for growth return [66]. In this study, we were able to observe that up to about 5% of akinetes were infected by their specific *R. akinetum* chytrid parasites. Some cyanobacterial filaments could thus be devoid of their storage cells [67]. In this way, we can suggest that the specific parasitism on akinetes could lower or delay the seasonal growth of cyanobacterial in succeeding year, although this hypothesis remains to be experimentally tested.

In addition to the above direct top-down impact on cyanobacteria communities, chytrid infection also affects the integrity of filaments and could reduce their size [68]. In a case of filamentous species, the cell-cell adhesion described by Flores and Herrero [69] is fundamental to the transfer of compounds essential for a healthy filament from cell to cell. By their parasitic action, chytrids kill their host cells. The death of cells inside a filament could induce the fragility of filaments, and impact the adhesion mechanism, which could result in the breaking of cyanobacterial filaments. This may help explain why, during our survey, the significant (p<0.001) reduction of the filament size which decreased from 52 to 10 cells filament^−1^ was significantly correlated with the increasing infection prevalence in the two sampling depths (rs = −0.79 and −0.84, p<0.001, for 0.5 m and at MF depth, respectively). Because the availability of filamentous cyanobacteria to grazing is mainly constrained by their inedible size [22], [23], we hypothesize that the “mechanistic fragmentation” of cyanobacterial filaments in small units by chytrid parasitism may increase the availability of cyanobacteria to grazers, and so, accelerate the decline of cyanobacterial blooms.

### Concluding Remarks

In this study, we were able to describe two apparent chytrid species with similar life cycles but with different strategies for infestion in the blooming filamentous cyanobacterial host *A. macrospora*, monitored using high sampling resolution in a productive freshwater lake. One chytrid species infected both vegetative cells and akinetes and was responsible for the death of cells within host filaments, while the other species infected only akinetes and may thus affect the survival of cyanobacteria hosts and their proliferation from year to year. We propose that with maximum prevalence levels, which increased from 4–6% of total cells and akinetes, 17% of total filaments, and >60% of total cells in infected filaments, chytrid parasitism is one of the driving factors involved in the decline of cyanobacterial blooms. In addition, by the so-called “mechanistic fragmentation” of cyanobacterial filaments, chytrids could weaken the resistance of *A. macrospora* to grazing, which could further accelerate the decline of blooms. This adds to the roles of chytrid zoospores which are well known to upgrade the biochemical diet of zooplankton [14], [70], establishing zoosporic fungi as potential key players in the food web dynamics. Our conclusions are empirical and mainly based on the results from field observations that require experimental validation. Furthermore, our study was conducted during a single bloom event and in one temperate lake. Clearly, although *A. macrospora* blooms are an annual event in Lake Aydat which we investigated, repeating a similar study for different bloom species, over several years, and on a wide geographical scale, remains necessary for accurate generalization.

## Supporting Information

Figure S1 **Nutrient concentrations during sampling period.** Ammonium (A), nitrate (B), and phosphorous (C) concentrations measured from the 6^th^ of September to the 30^th^ of October 2010 at 0.5 (dark dashed line) and MF (grey dashed line) depths.
(TIF)
