# Supplementary figures and images for: Fungal Parasitism: Life Cycle, Dynamics and Impact on Cyanobacterial Blooms
Source: PLoS One. 2013 Apr 12;8(4):e60894. doi: 10.1371/journal.pone.0060894 (PMC3625230; doi:10.1371/journal.pone.0060894)

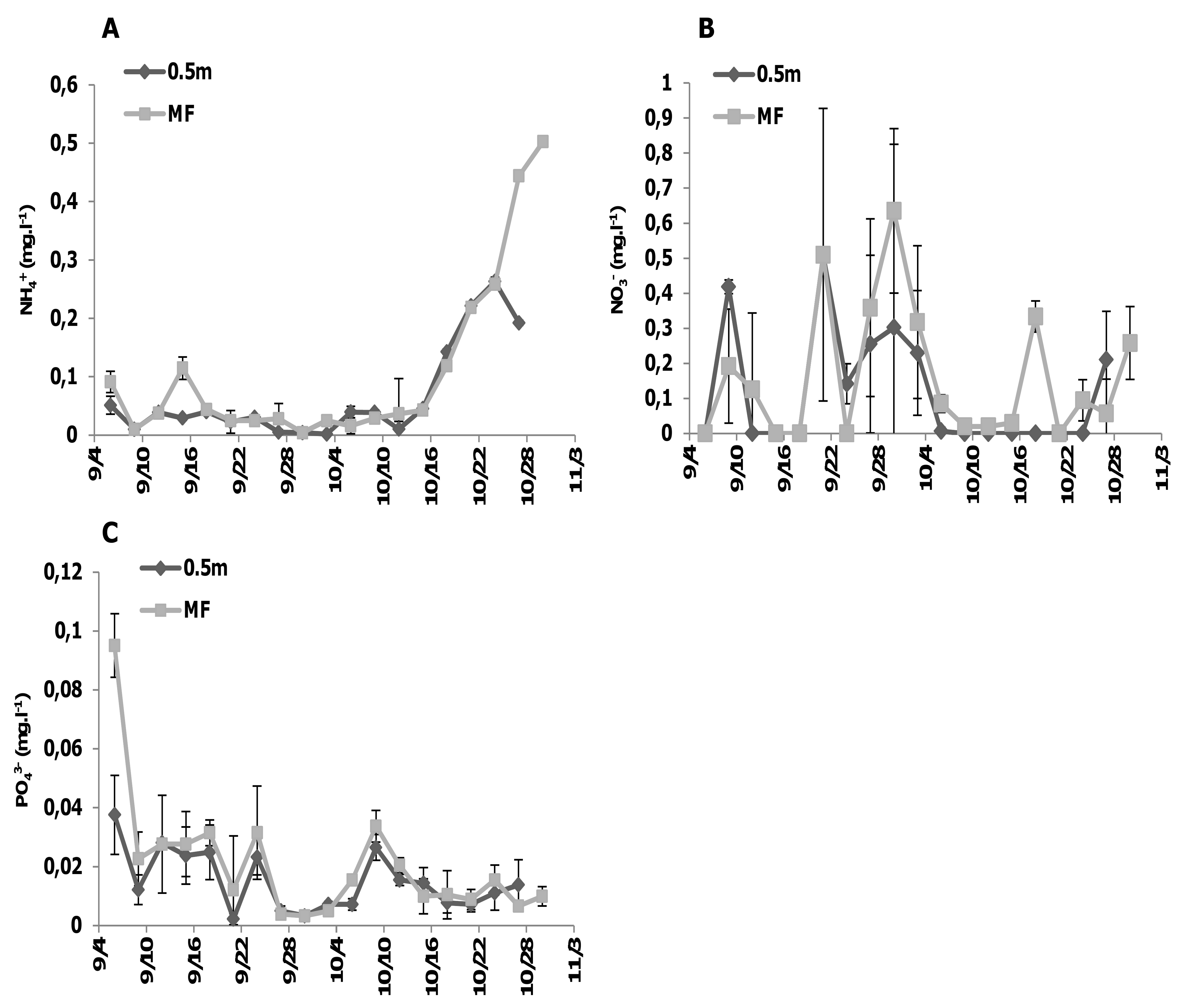

Supplement: Figure S1 — Nutrient concentrations during sampling period. Ammonium (A), nitrate (B), and phosphorous (C) concentrations measured from the 6th of September to the 30th of October 2010 at 0.5 (dark dashed line) and MF (grey dashed line) depths. (TIF) [file pone.0060894.s001.tif]
